# Factors influencing physicians’ choice of workplace: systematic review of drivers of attrition and policy interventions to address them

**DOI:** 10.7189/jogh.06.020403

**Published:** 2016-12

**Authors:** Maria El Koussa, Rifat Atun, Diana Bowser, Margaret E Kruk

**Affiliations:** 1 Harvard T.H. Chan School of Public Health, Harvard University, Boston MA, USA; 2 The Heller School for Social Policy and Management, Brandeis University, Waltham, MA, USA

## Abstract

**Objectives:**

The movement of skilled physicians from the public to the private sector is a key constraint to achieving universal health coverage and is currently affecting health systems worldwide. This systematic review aims to assess factors influencing physicians’ choice of workplace, and policy interventions for retaining physicians in the public sector.

**Methods:**

Five literature databases were searched. Studies were included in the review if they focused on at least one of the following criteria: (i) incentives or motivators for retaining physicians in the public sector, (ii) pull factors that encouraged physicians to move to the private sector, (iii) push factors that forced physicians to leave the public sector, (iv) policy interventions or case studies that addressed physician retention in the public sector, and (v) qualitative reviews of policy interventions that were implemented in different health system settings.

**Results:**

Nineteen articles met the inclusion criteria. Six major themes that affected physicians’ choice of workplace were identified including: financial incentives, career development, infrastructure and staffing, professional work environment, workload and autonomy. The majority of the studies suggested that the use of financial incentives was a motivator in retaining physicians in the public sector. The review also identified policy interventions including: regulatory controls, incentives and management reforms. Regulatory controls and incentives were the two most frequently reported policy interventions.

**Conclusion:**

While factors affecting physicians’ choice of workplace are country specific, financial incentives and professional development are core factors. Other factors are highly influenced by context, and thus, it would be useful for future cross–country research to use standardized data collection tools, allowing comparison of contextual factors as well as the examination of how context affects physician retention in the public sector.

The World Health Organization (WHO) estimates that in order to expand universal health coverage, the world needs an additional 12.9 million skilled health professionals by 2035–physicians, nurses and midwives [1]. Skilled health professionals are needed to achieve, maintain and accelerate progress on universal health coverage by ensuring effective coverage for an expanding set of health care needs for all populations [1]. The human resource shortages are particularly acute in low and middle income countries in Africa, Asia and the Pacific and exacerbated by the movement of skilled physicians from the public to the private sector affecting health systems worldwide [2,3].

In both low– and high–income countries, physicians working in government hospitals and clinics also often practice privately in order to boost their earnings. This dual practice is common in many European countries including the United Kingdom and is particularly high in low– and middle–income countries such as Egypt, Vietnam and India [4]. The income gap between the public and private sector in these countries is a key factor motivating physicians to leave the public sector or work in both the public and private sector. Indeed, it is increasingly uncommon to find full–time health workers who are civil servants exclusively working in the public sector [4–7]. For instance, in Austria approximately 100% of senior health specialists work in both sectors, in the United Kingdom 60% of public physicians work in both sectors [8]. In Ireland, more than 90% of physicians employed in public hospitals also have privileges to practice in the private sector [8].

Other factors that have been identified as driving the movement of physicians from the public to the private sector include: lack of academic and career development opportunities in the public sector, poor infrastructure in public facilities, and greater autonomy in the private sector [2,9]. The pervasive practice of dual practice and the shift of doctors from the public sector to the private sector suggests a need to reassess the traditional models of physician education, placement and compensation, and the functioning of labor markets for highly skilled health workers [1]. There is limited evidence on the policies and regulatory mechanisms for promoting physician retention in the public sector [8,9].

The aim of this systematic review is to assess factors influencing physicians’ choice of workplace and potential policy interventions for retaining physicians in the public sector. The review will identify the sources of dissatisfaction of physicians in the public sector (push factors), and sources of satisfaction of physicians in the private sector (pull factors), as well as the advantages and disadvantages of different policy interventions addressing physician retention in the public sector. The analysis will inform policymakers on the current evidence and identify policy options for retaining physicians in the public sector.

## METHODS

The analysis was undertaken by the first author and lead author. At each step of the process, the first author independently assessed the eligibility of individual studies. Results were shared with the lead author, and differences were reconciled through discussions.

### Identification of studies

We conducted a systematic review of factors and policies related to attrition and retention of physicians in the public sector. Using key search terms, the following literature databases were searched: PubMed, EMBASE, World Bank database, WHO Global Health Observatory as well as Google Scholar. The search terms included: “physicians”, “doctors”, “specialists”, “retention”, “dual practice”, “recruitment”, “incentives”, “motivation”, “retaining”, “motivating”, “physician incentive plans”, “public sector”, “public health sector”, “private sector”, and “private health sector”. The references of the included studies were also scanned in order to find relevant literature that was not identified through the database search.

### Study selection criteria

Studies were included in the review if the original research was presented and if the study population included physicians. The studies also focused on at least one of the following criteria: (i) incentives or motivators for retaining physicians in the public sector, (ii) pull factors that encouraged physicians to move to the private sector, (iii) push factors that forced physicians to leave the public sector, (iv) policy interventions or case studies that addressed physician retention in the public sector, and (v) qualitative reviews of policy interventions that were implemented in different health system settings. Studies were excluded if they were: not in English, published before 1980, non–human studies, book chapters or book reviews, dissertations or theses, or published abstracts. Studies were also excluded if they were focusing on physician retention in rural vs urban areas since the review will be focusing only on public vs private retention, and if they were discussing retention of health workers other than physicians such as nurses or midwives.

### Data extraction and analysis

The studies were classified according to income level based on the World Bank classification. The study characteristics were extracted from all the studies and these include: first author, year, countries involved, study design and method (**Table 1**). The studies were then grouped into two categories: studies that were focusing on factors affecting physicians’ choice of workplace (push–pull factors), and those that were discussing policy interventions for retaining physicians in the public sector.

**Table 1:** Studies included in the systematic review

Income level	First author	Year	Country	Study Design	Method
Low and low–middle income	Abdul Rahim [2]	2012	Multiple countries	Descriptive study	Evaluation of five worldwide policy initiatives
	Luboga [10]	2011	Uganda	Mixed–method study	Focus groups and questionnaires
	Malik [11]	2010	Pakistan	Mixed–method study	Open ended questions, questionnaire and interviews
	Russo [12]	2014	Cape Verde, Guinea Bissau and Mozambique	Mixed–method study	Qualitative interviews and surveys
	McPake [13]	2014	Mozambique, Guinea Bissau and Cape Verde	Cross–sectional study	Survey
	Lonnroth [14]	1998	Vietnam	Qualitative study	Individual interviews and group discussions
	Gruen [15]	2002	Bangladesh	Qualitative study	Open–ended questionnaire and in–depth interviews
	Jan [16]	2005	Multiple countries	Descriptive study	Critical analysis of dual practice policies
Upper middle and high income	Ashmore [3]	2013	South Africa	Qualitative study	Qualitative interviews
	Ashmore [17]	2015	South Africa	Qualitative study	In–depth interviews
	Ashton [18]	2013	New Zealand	Cross–sectional study	Postal survey
	Andreassen [19]	2013	Norway	Prospective cohort study	Modeling physicians labor supply choices
	Longmore [20]	2014	South Africa	Qualitative study	Open–ended questionnaire
	Gonzalez [21]	2004	Not listed	Modeling	Principal–agent modeling
	Heponiemi [22]	2013	Finland	Prospective cohort study	Four–year prospective questionnaire study
	Cohn [23]	2009	United States	Case–study	Case study on the journey of Banner Medical Group
	Kankaanranta [24]	2007	Finland	Retrospective cohort study	National postal survey completed at 5 y intervals
All income levels	Gonzalez [8]	2013	Not listed	Modeling	Two–stage theoretical modeling
	Eggleston [4]	2006	Not listed	Descriptive study	Comparative analysis of five models of dual practice

**Figure 1:**
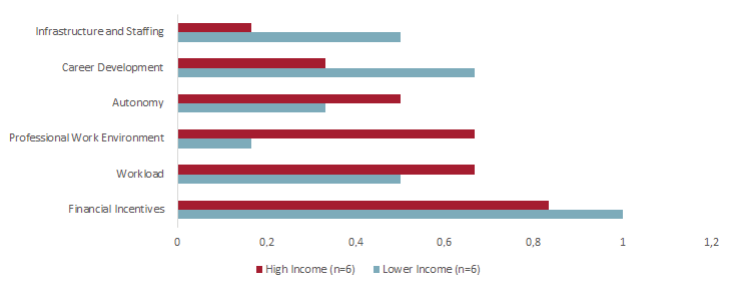
Proportion of studies by income category discussing themes affecting physician’s choice of workplace.

Thematic synthesis was adopted in our review as codes were generated from illustrative quotes and then classified into themes [25]. During the data extraction process, the first author extracted illustrative quotes on push and pull factors from the studies focusing on physicians’ choice of workplace. These quotes were coded according to trends in common answers, and sub–themes/factors were generated, 15 factors in total (**Figure 1** and **Table 2**). The factors were then synthesized into six main themes, including: financial incentives, career development, infrastructure and staffing, professional work environment, workload, and autonomy. The results and discussion sections were organized according to those themes. Each study can include more than one push–pull factor and is counted more than once when calculating the proportions in the results section (**Figure 1** and **Table 2**).

**Table 2:** Factors affecting physician’s choice of workplace

Theme	Sub–theme/factor	First author’s last name	Push factors (illustrative quotes)	Pull factors (illustrative quotes)
**Financial incentives**	Adequacy of financial compensation	Ashmore [3], Luboga [10], Malik [11], Andreassen [19], Lonnroth [14], Gruen [15], Ashmore [17], Russo [12], McPake [13]	“**None** of the physicians in our focus group discussions felt their **compensation** was acceptable” [10]. “The **most frequently** mentioned conditions by those who would consider leaving government services were: **payment of a compensation for transition** (n = 18); a social pension scheme (n=13); tax relief (n=7); and credit offers (n=6)” [15]. “**Median income** among physicians in the public sector was significantly **lower** than that of physicians in the private sector (*P*<0.001) and dual practice (*P*<0.001), but income differences between private–only and dual practice physicians were not statistically significant” [12].	“Some specialists interviewed appeared to value **high financial rewards** more than others, and thus felt more desire to work in the private sector” [3]. “… physicians in private setups were motivated by the **availability of financial incentives** other than pay and good working conditions in their current jobs” [11]. “Our study implies that **overall wage increases and tax reductions** give the medical doctors an **incentive** to move to full time jobs, in particular in the private sector, at the expense of working in other jobs in the health sector of the economy” [19]. “In some districts, physicians are on the payroll, but may come to work only half the day, largely because they are attending to private practices as a means of **income supplementation**” [10]. “When asked about the reasons for engaging with the private sector, answers from the survey were broadly consistent with the qualitative findings, with most physicians reporting **“increasing income”** as the main factor for practicing in the private sector (95.5% responding important or very important)…” [12].
	Income relative to workload	Ashton [18]		“… the private sector is valued for the opportunity to work independently (4.45), the freedom to apply ideas in the workplace (4.28) and the **income earned relative to the workload** (4.06).”
	Sustainability of income	Lonnroth [14]	“All interviewees expressed **difficulties in living on the salary** in the public health care sector” [14].	“A significant finding was that all the non–private physicians said that they had previously tried to go private or would try to go private if they **could not support themselves** or their family financially” [14].
	Financial security	Longmore [20], Luboga [10]	“There was much emotion surrounding remuneration inconsistencies and resulting **financial insecurity**, doctors feeling that it is simply not acceptable to **fail to pay salaries on time…**”[20]. [20] “The other problem is **job security**, in most cases you don’t know where you will go, they can fire you at any time…” [10] [10].	
**Career development**	Professional development	Ashmore [3] [3], Luboga [10] [10], Malik [11] [11], Gruen [15] [15]	“There was also **dissatisfaction** expressed in the public sector with the sense of **career progression**. It was repeatedly noted how once a senior specialist in the public sector, it is easy to become ‘stuck’, for example, since there are few chief or principal specialists jobs available” [3]. “Only about **one fourth** of physicians (26%) said their employer offered **sufficient opportunities for promotion**” [10].	“There was also a definite sense that the private sector presented opportunities for more recognition of one’s experience and seniority, and thus **a sense of career progression**, if only through higher prestige and, relatedly, higher wages.” [3] “Most of the participants intended to change their current position (86%), mainly for **professional development** (66%) and better income (21%).” [15]
	Education and training opportunities	Luboga [10] [10], Gruen [15] [15]	“More than 66% of the doctors in primary and secondary care considered **training opportunities to be poor**, as opposed to 33% of the doctors in tertiary care facilities” [15].	“A sizeable number (66%) rated **“access to higher education”** as “very important”, and another large portion (60%) said this was an important enough issue for which to consider changing jobs” [10].
	Physician reputation	Russo [12] [12], Kankaanranta [24] [24]		“For dual practitioners, the main motivations were opportunities to increase income, to **consolidate professional reputation**, and to take advantage of the complementarities between the two job modalities” [12]. “Also, ***Generally held to be a prestigious position*** seemed to have high t–test values each year, implying that it is also a good indicator of physician’s job satisfaction, when the variable *Good income compared to workload* was modelled as a base variable” [24].
**Infrastructure and staffing**	Resource availability	Ashmore [3], Luboga [10],, Malik [11], Gruen [15]	“On the other hand, the public sector was noted to have **fewer resources and less equipment and drugs available**, factors which hindered the ability to do one’s job as desired, often considered frustrating” [3]. “There are significant problems with working conditions in all health facilities. **Access to equipment, supplies, drugs, electricity, and water are seriously compromised**” [10]. “In contrast, demotivators in current jobs were mostly organizational factors including fewer opportunities for higher qualifications, **resource unavailability** and poor supervision” [11].	“So at least the other advantage of being in the private sector [is] you get to see **what’s current and what’s currently in use as well**, which we don’t have on the other side.” [3] “Physicians (and other health workers) in the private (non–profit) sector were more likely to rate working conditions, more highly, with statistically significant differences measured for the **availability of supplies, equipment and drugs, utilities**, transportation, and time for workers to eat lunch.” [10]
	Staffing shortages	Ashmore [3], Luboga [10], Gruen [15]	“**Lack of public sector staff, relative to the private sector**, was another resource issue that caused public sector dissatisfaction” [3]. “**Lack of doctors themselves** also caused dissatisfaction, implying there is a damaging cycle where retention is a problem, since lack of retention may encourage others to leave” [3]. “Physicians discussed **staffing shortages**, unreasonable patient loads lack of available specialists, and **positions that have gone unfilled for months or even years**” [10].	
	Working conditions	Luboga [10], Malik [11], Gruen [15]	“Physicians in five of eight focus group discussions complained of **infrastructure issues**, complaining about a lack of clean water or electricity, not enough beds for patients or space in the wards, and poor infection control” [10]. “Conversely, physicians working in primary care health facilities more often reported **poor working conditions** as a demotivator” [11]. “The most often agreed opinions referred to lack of drugs and equipment (22%), long waiting times (17%), lack of doctors and nurses (13%) and **lack of cleanliness** in government facilities (11%)” [15].	“Physicians (and other health workers) in the private (non–profit) sector were more likely to **rate working conditions, more highly**, with statistically significant differences measured for the availability of supplies, equipment and drugs, utilities, transportation, and time for workers to eat lunch” [10]. [10] “Conversely, physicians in private setups were motivated by the availability of financial incentives other than pay and good working conditions in their current job” [11].[11]
**Professional work environment**	Relationship with patients	Ashmore [3]	“Patient relationships also seem to be **strained** in the public sector, due to relative unwillingness or inability of patients to follow directions, as well as, potentially, some classism and racism among doctors” [3].	
	Relationship with supervisors and administration	Ashmore [3], Luboga [10], Longmore [20], Kankaanranta [24]	**“Distrust of the public hospital ‘administration’ and DoH**, meanwhile, seemed universally high.”; “The above respondents were noticeably embittered towards state and hospital management, which seemed almost universal” [3]. “In questionnaires, physicians were the **least likely** to say their immediate supervisor (presumably, upper management) **“cares about me as a person”**, and the **least likely** to say they received **recognition** for doing good work” [10]. “Sixty–four per cent of doctors felt that they were **not respected and valued by HR staff**” [20].	“Whatever the reasons, in H1 at least, relations **between different health providers** (rather than between doctors) were generally perceived as **much better** in the private sector” [3]. “In one focus group at a private facility, physicians spoke of **supervisors who respected staff, assisted in problem solving, and instilled a sense of ownership and responsibility in staff**” [10].
	Managerial interference	Ashton [18]	“Key sources of dissatisfaction were workload pressures, mentally demanding work and managerial inferences” [18].	“They also have a good income relative to their workload and little managerial interference” [18].
**Workload**	Work hours, amount of work and workload pressures	Ashton [18], Luboga [10], Malik [11], Russo [12], Kankaanranta [24]	“While our survey did not include questions specifically related to levels of stress, dissatisfaction was higher in the public sector for all sources of dissatisfaction. These included factors related to stress such as poor employer/employee relations, **workload pressures and mentally demanding work**” [18]. “Only about a third (36%) of physicians said they thought their workload was manageable. All focus group physicians **complained about work overload**” [10]. “In public setups, tertiary physicians reported **long duty hours**, less personal safety and **heavy workloads** as important demotivators compared with those in private setups…” [11]. “For factors affecting *job dissatisfaction*, variables such as **Tight, inflexible timetable**, *Poor employee/supervisor relations*, and *Tense atmosphere in workplace* had the highest t–test values, when the variable ***Monotonous work*** was modelled as baseline” [24].	“For those working exclusively in the private sector the motivations were higher earnings, autonomy, and **flexibility of working hours**” [12].
**Autonomy**	Ability to apply their own ideas and flexibility in patient treatment	Ashton [18], Ashmore [3], Russo [12], Lonnroth [14], Kankaanranta [24]	“The ability to work with more autonomy in the private sector, however, did appear to carry a distinct advantage for those who valued it. This seemed particularly true of those **frustrated with public ‘regulations and rules’**, who wanted to work on their own terms” [3]. “Complicated procedures in the public sector mentioned by the interviewees include **‘bureaucratic’ procedures** to fulfill eligibility criteria for free or subsidized treatment as well as **rigid** diagnostic and treatment strategies that follow more or less **fixed guidelines**” [14].	“…**work autonomy and flexibility** are the key motivations at the base of their choice to dedicate exclusively to the private sector, since earnings are not significantly different from those of dual practice physicians” [12]. “In contrast, when working in the private practice, specialists value the opportunity to work **independently** and to **apply their own ideas** in the workplace” [18]. “Private physicians on the other hand can apply **more flexible** approaches to **diagnostic procedures and choice of treatmen**t, which are influenced by patients’ preferences and ability to pay” [14]. “Both private and non–private physicians said that private practitioners provided more **flexible and individualized care**, which they described as appealing to patients” [14]. “Each year, the variable ***Chance to apply one’s own ideas in the work*** emerged as one of the most important job satisfaction dimensions affecting intention to change work sector” [24].

**Figure 2:**
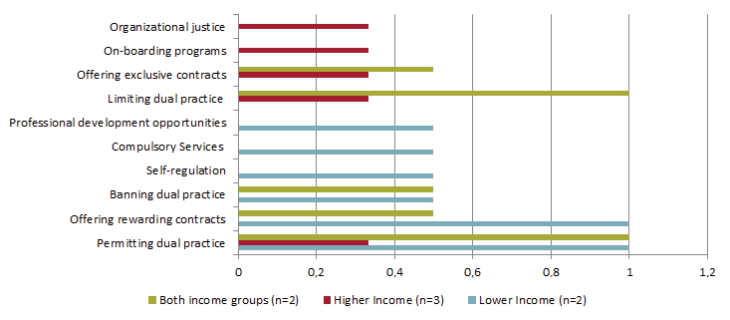
Proportion of studies by income category discussing policy interventions to address physician retention in the public sector.

For the policy intervention studies, illustrative quotes were pulled for the advantages and disadvantages of each policy intervention. These policy interventions were then synthesized into three main themes. Each study could include advantages and disadvantages for more than one policy intervention and was counted more than once when calculating the proportions in the results section (**Figure 2** and **Table 3****)**.

**Table 3:** The advantages and disadvantages of policy interventions for addressing physician retention in the public sector

Policy themes	Policy interventions	First author	Disadvantages (illustrative quotes)	Advantages (illustrative quotes)
**Regulatory controls**	Banning dual practice	Gonzalez [8], Jan [16]	“The **more able** ones tend to be more involved in the **private sector** since their ability allows them to get a higher return. The **less able** tend to combine both public and private activities if dual practice is allowed, or work only in **public practice** when this is not the case. When dual practice is forbidden, the **population of physicians** working the public sector for a given salary **decreases**” [8]. “In addition, when the public and private sectors do not share physicians, higher private sector earnings are expected to attract more highly skilled physicians, leaving those of **less ability in the public sector**” [8]. “In practice, bans do not prevent these activities, but instead take them **outside the regulatory and policy jurisdiction** of government” [16].	
	Permitting dual practice	Gonzalez [21], Eggleston [4], Jan [16], Abdul Rahim [2], Gonzalez [8]	“We found that the physician’s dual practice has conflicting effects. On the one hand, his interest in curing patients and gaining prestige, generates an **over–provision of health services**” [21]. “These theoretical predictions stand at odds with much of the policy discussion, which tends to assume that allowing public sector physicians to earn private revenue will harm the quality of services provided in the public sector, although it may **benefit private sector patients and physicians**” [4]. “Since monitoring of provider time and effort is costly, often only minimal presence in a public practice is required to access the non–pecuniary benefits of public employment (eg, official salary and civil servant fringe benefits such as public housing)” [4]. “A physician with both public and private practices may **use public resources to treat private patients**, whether by lifting supplies (eg, gauze, medications) or treating patients at the public facilities without paying any rent or charge for such use” [4]. “Furthermore, dual practice providers may have incentives to induce demand for private practice services. The propensity of health care providers to **over–refer** to facilities in which they have financial interest is widely recognized” [4]. “However, dual job holding by public sector health professionals is potentially a problem because it may create inappropriate incentives as the **boundaries** between a public health professional’s day–to–day job and his or her private practice **can become blurred**” [16]. “Firstly, it can encourage the **misappropriation** of scare **public sector resources** into the private sector” [16]. “The second reason why private practice by public health workers has been posited as a problem is because it may lead to doctors **diverting patients from public facilities into private services**” [16]. “Also, **no evidence** thus far supports dual practice as a method of **improving equitable delivery of healthcare**” [2].	“On the other hand, if the HA is able to control these incentives to over–provide services, then it can benefit from the physician’s increased interest in doing **more–accurate diagnosis**” [21]. “Interestingly, some consistent results emerge from these diverse conceptualizations: (1) allowing dual practice may i**mprove social welfare**; and (2) allowing dual practice may improve the **quality of public services**, under specific circumstances” [4]. “Allowing dual practice may enable the government to recruit **quality providers** at a **modest budgetary expense**” [4]. “To the extent that physicians attempt to build a good reputation that will enhance future private practice revenue, allowing dual practice also gives a kind of **performance–based incentive** for physicians to exert effort” [4]. “From the point of view of the public sector, allowing health professionals to engage in private practice can be a means of **minimizing the budgetary burden required to retain skilled staff**” [16]. “In contrast to these measures, the potential value of recognizing and legitimizing dual practice is that, at one level, it enables some **degree of control** to be exercised over **quality and safety**” [16]. “The importance, therefore, of providing official recognition is that it allows policy–makers to incorporate such activity within the bounds of its **regulatory and policy jurisdiction**” [16]. “Practitioners would continue to enjoy the **prestige of public sector** positions and **ongoing career development** while mitigating **economic opportunity costs** otherwise incurred if solely servicing the government...” [2]. “This implies that dual practice might be desirable because it allows the HA to **reduce the wage needed to retain** physicians working in the public sector” [8].
	Limiting dual practice	Gonzalez [8], Gonzalez [21], Eggleston [4]	“Overall, **profit limitations** have a **milder effect** on the amount of **dual practice** performed by physicians.” [8]. “Secondly, focusing on limiting policies, we have shown that **limiting income** is always **less effective** than **limiting involvement**” [8]. “Therefore, our conclusion is that this sort of regulatory policy may be beneficial from a social point of view, although it can generate as a non–desired effect a **reduction** on physicians’ **incentives** to perform **accurate diagnoses**” [21]. “In either case, the limits on dual practice o**nly affect behavior** if physicians anticipate that the **contractual terms will be enforced**” [4].	“… as it only affects the **high skilled** physicians that are compelled to r**educe private involvement** in order to satisfy their earning constraint” [8]. “In contrast, policies that limit involvement directly target the intensity of dual practice and are therefore **more effective in limiting its costs**” [8]. “We have shown that if physicians’ payment contracts include **proper incentives**, then **limiting** physician’s **private income** can be **optimal**, whereas introducing exclusive contracts is always useless” [21]. “Better ability to monitor and contract can **minimize shirking on public practice duties, appropriating supplies and using public equipment without paying rent**. Transparent contractual relationships between public and private practices, such as rental of facilities and subcontracting for specific services, can **offset many of the costs** associated with allowing the same physicians to practice in both” [4].
	Self–regulation	Jan [16]	“Indeed, in certain circumstances, this could lead to an **incentive to “overprovide” quality in the public sector**, particularly, in high–income settings, because the health facility rather than the individual doctor bears the cost of providing additional quality” [16]. “Consequently, there is a certain trade–off between quality and access to health care because **higher–quality services** will tend to be **more costly**, and thus specific measures addressing **financial access** need to be **considered** when proposing such forms of self–regulation” [16].	“Self–regulation of this nature works because significant weight is given to an individual’s **reputation** as a doctor in public practice, which influences his or her **income–generating** capacity in **private** practice” [16]. “The role of such regulation could be viewed as **addressing the uncontrolled proliferation** of private providers and, in a sense, establishing **barriers to entry**” [16]. “The rationale for professional self–regulation is that it **recognizes the collective interest** in instituting some form of cooperative behavior among individual agents” [16].
	Compulsory services	Abdul Rahim [2]	“The **evidence** base on effectiveness of compulsory services to date remains **lacking**” [2].	
**Incentives**	Offering exclusive contracts	Eggleston [4], Gonzalez [21], Gonzalez [8]	“The problem with this measure is that in the context of the strict resources constraints that often exist within low– and middle–income countries, such payments can be prohibitively **costly**–particularly if incomes in the private sector are high and thus there is a **need for greater levels of compensation**” [4]. “We have shown that if physicians’ payment contracts include proper incentives, then limiting physician’s private income can be optimal, whereas introducing **exclusive contracts is always useless**” [21].	“Exclusive contracts, however, are shown to be a **useful tool for cost–containment** when physicians are paid on **a salaried basis**” [21]. “This illustrates how exclusive contracts offer **greater flexibility** for the HA to **mitigate loss of productivity** associated with dual practice, which makes the HA less interested in banning dual practice when rewarding policies are available” [8].
	Offering rewarding contracts	Gonzalez [8], Abdul Rahim [2], Jan [14]	“Rewarding policies, ie, those that pay an extra amount to physicians who give up their private practice, are **only desirable when limitations are difficult to enforce**” [8]. “The Commission determines salaries for public sector workers and hence deems it **unfair to selectively raise wage of health employees and exclude other sectors**” [2]. “The most immediate and overriding **constraint on the feasibility** of this option however is the **cost** to the **public** sector. In circumstances where there are tight resource constraints in the public sector, this option is unlikely to be feasible” [14].	“Remuneration should reflect the level of work responsibility and be deemed fair vis–à–vis other sector counterparts to ensure continued **attraction and retention** of staff” [2]. “Furthermore, a **mix of payment mechanisms** such as time–based, service–based and population–based is linked with **enhanced provider performance**” [2]. “On this basis, the incentive to **shift** effort from **public–sector to private–sector** work would be **offset by making remuneration for public practice**, like that of private practice, related to effort or output” [14].
	Providing professional development opportunities	Abdul Rahim [2]		“Continued education, interactive training and professional development geared towards the priority health conditions and needs of the local population improves health worker **competency and motivation**” [2].
**Management reforms**	On–boarding programs	Heponiemi [22]	“Organizational justice was **not able to buffer** the association between being or becoming a new public GP and **turnover intentions**” [22]. “Our results showed that new public GPs had 2.6 and those who stayed as public GPs both times had 1.6 times **higher likelihood of having turnover intentions** compared to those who stayed at other positions both times” [22].	“Our results suggest that by improving organizational justice primary care organizations could **improve** GP’s job **satisfaction** and involvement and consequently maybe increase GP work’s **attractiveness as a career option**. For example, organizations could **invest in supervisor training**, particularly because previous studies have shown that leaders can be trained to act in a more just manner and this in turn improves subordinates’ attitudes and behavior” [22].
	Organizational justice	Cohn [23]		“One year after the on–boarding program was initiated, **not a single new physician left** BMG, which is a sharp turnaround from the 10 percent loss the group experienced previously” [23]. “Since the onboarding program began, however, everyone who has worked with the new physicians (including allied health professionals) has noted an **improvement in physician morale** and in the **practice environment**” [23].

## RESULTS

**Figure 3:**
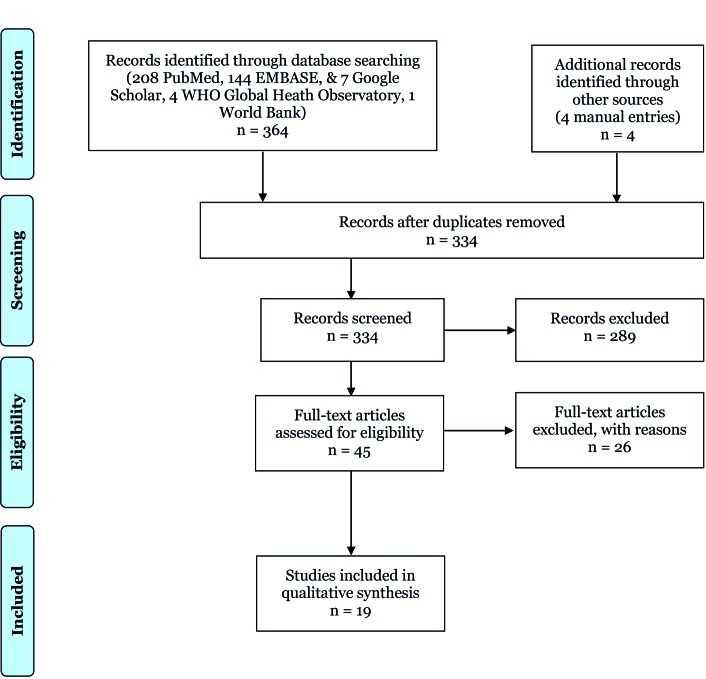
Flowchart on the database and the study selection.

The database search identified 368 hits (208 PubMed, 144 EMBASE, 7 Google Scholar, 4 WHO Global Health Observatory, 1 World Bank, and 4 manual entries) (**Figure 3**). Using the study selection criteria, the titles and abstracts were screened and 45 articles remained. After the full–text examination of the articles, 19 articles were included in the review. **Figure 3** uses the PRISMA framework to show the flow of the search during the different stages of analysis [26].

**Figure 4:**
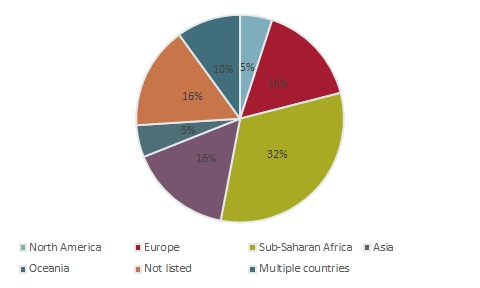
Geographic origin of included studies (n = 19).

Decisions to include or exclude studies were made by the first author under the supervision of the lead author. The countries studied were from Sub–Saharan Africa (South Africa, Uganda, Cape Verde, Guinea, Mozambique and Bissau), Europe (Norway and Finland) Asia (Pakistan, Vietnam, Bangladesh, and Malaysia), North America (United States) and the Pacific (New Zealand) (**Figure 4** and **Table 1***)*. The review included 12 studies on push–pull factors for physician retention and 7 studies on policy interventions for retaining physicians in the public sector.

### Factors affecting physicians’ decision to leave public sector/enter private sector

We identified six main themes that affected physicians’ choice of workplace including: financial incentives, career development, infrastructure and staffing, professional work environment, workload and autonomy (**Table 2**).

The majority of the studies (number n = 11; 92%) highlighted the importance of financial incentives in determining physicians’ choice of workplace [3,10–15,17–20]. Inadequacy of financial compensation and financial insecurity were found to be major factors that encouraged physicians to leave the public sector or practice in both the private and public sector (dual practice). Competitive salaries and higher income were the main reasons physicians were motivated to move to the private sector [3,10–15,17–20]. In one of the studies that focused on three lower income cities in sub–Saharan Africa, it was found that 95.5% of physicians reported increasing income as an important or very important factor in their decision to practice in the private sector [12].

Career development was also an important motivator and was discussed in around 50% (n = 6) of the studies [3,10–12,15,24]. One study in Uganda found that only one out of four physicians in the public sector reported that their employer offered them sufficient opportunities for promotion [10]. In the same study, a significant number of physicians (66%) ranked access to higher education as very important, and 60% of physicians reported that this was a significant issue that would lead them to consider changing jobs [10]. Physicians reported the lack of opportunities for professional and academic development, stagnant career progression and poor training opportunities as reasons that would push them to leave the public sector or practice in both the public and private sector. Opportunities for professional development, and being in a prestigious position were identified as main factors that were pulling physicians into the private sector in some of the countries [10–12,15,24]. On the other hand, in countries like South Africa and New Zealand where central hospitals are run by public academic institutions, physicians working in the public sector are presented with professional development opportunities [3,18]. These include free use of research and other academic facilities, and greater opportunities for teaching and research [3,18].

Infrastructure and staffing were highlighted in 33% (n = 4) of the studies as being important determinants of choice of work place [3,10,11,15]. Lack of resources, shortage of staff, unfilled physician positions, poor facility infrastructure and poor working environment were important push factors in the public sector. The availability of up–to–date resources and positive work environment were motivating factors that were pulling physicians to work in the private sector or both in the public and private sectors [3,10,11,15].

Approximately 42% of the studies (n = 5) [3,10,18,20,24] indicated that professional work environment plays an important role in retaining physicians in the public sector. Lack of trust with the hospital administration team, poor patient–physician relationships, high managerial interference, lack of respect and appreciation of physicians, and poor supervisor–employee relationships were the main push factors reported in the studies. The pull factors reported in the studies were: positive relationships between physicians, stronger patient–physician relationships, low managerial interference, and strong supervisor–physician relationship [3,10,18,20,24].

Approximately 58% of studies (n = 7) mentioned workload and autonomy as important factors in physician retention [3,10–12,14,18,24]. Push factors that were mentioned include: workload pressures, mentally draining work, heavy workload, long hours, low autonomy, bureaucracy and rigidity in patient treatment and inflexible schedule. The pull factors that attracted physicians to the private sector were: high autonomy, the ability to apply their own ideas and flexibility in diagnostic treatment and procedures [3,10–12,14,18,24].

### Policies for retaining physicians in the public sector

The review identified a range of policy interventions that focused on improving physician retention in the public sector along with their advantages and disadvantages. Of the seven studies included two discussed options for policy interventions in lower income countries, three focused on higher income countries and two compared policy interventions in both lower and higher income countries (**Figure 2**). We found three main categories of policies for retaining physicians in the public sector: (a) regulatory controls, (b) incentives and (c) management reforms (**Table 3**).

(a) Regulatory controls included banning dual practice, permitting dual practice, limiting dual practice, professional self–regulation and compulsory services. Around 72% of the studies (n = 5) highlighted regulatory controls as policy interventions to address physician retention in the public sector [2,4,8,16,21]. The studies did not identify advantages for banning dual practice, however, disadvantages included: the increased likelihood for highly skilled physicians to move to the private sector, and the risk of having physicians practicing in both sectors illegally [8,16].

In terms of permitting dual practice, the following advantages were reported: improvement of the quality of service in the public sector, increased interest of physicians to provide more accurate diagnosis, establishment of performance based incentives, enhanced ability to retain skilled physicians without a budgetary burden, and increased possibility of exercising a higher degree of control over quality and safety. As for the disadvantages these include: the possibility of over–provision of medical services, the potential harm to the quality of services in the public sector, utilization of public resources in private practice, over–referrals, lack of clear boundaries between the public and private sector, lack of evidence showing improvement of equitable health care delivery, and higher time allocation in private practice while taking advantage of employment benefits in the public sector [2,4,8,16,21].

The studies highlighted several advantages for limiting dual practice such as: effectiveness in reducing costs on the supply side, encouragement of highly skilled physicians to reduce private practice involvement, and high efficacy when physician contracts include incentives and offsets costs associated with dual practice. There are also disadvantages in limiting dual practice including: reduction in physicians’ incentives to provide accurate diagnoses, and limited efficiency of this intervention if contractual terms were not enforced [4,8,21].

Professional self–regulation, was identified as an important function that provided the opportunity to introduce higher standards among practicing physicians, which in turn helped to enhance the prestige among physicians who met established standards [16]. Self–regulation could also improve quality of physician services in the public sector, and establish de facto barriers to enter the private sector by controlling licensing or certification [16]. Compulsory public sector service was also noted as a potential policy intervention [2]. A disadvantage of this policy is the lack of studies that assess the effectiveness and success of compulsory services in retaining physicians in the public sector [2].

(b) Incentives for retention in the public sector included: exclusive contracts, offering rewarding contracts and/or financial incentives, and providing professional development opportunities. Around 72% of the studies (n = 5) highlighted incentives as policy interventions to address physician retention in the public sector [2,4,8,16,21]. Exclusive contracts were identified as being useful for salaried physicians, but noted to be expensive to implement and not be as useful when physicians had incentive contracts [4,21].

In terms of rewarding contracts and financial incentives, the advantages of this policy intervention were the attraction and retention of physicians in the public sector, the enhancement of physician performance, and the reduction in the loss of productivity in the public sector. As for the disadvantages, these included: costliness in some cases, limited feasibility and it is only considered when limitations are difficult to impose [2,8,16]. For example, Jan et al. stated that the cost to the public sector was the main obstacle that prevented the use of incentives as a policy option, particularly in situations where there are resource constraints in the public sector [16]. Gonzalez and Macho–Stadler also reported that policies that provided financial incentives for physicians who leave the private sector were only appealing when limitations on private practice could not be imposed [8].

Provision of professional development opportunities was also listed as an intervention that motivated, and improved the competency of physicians in the public sector. No disadvantages were identified, however [2].

(c) Management reforms identified in the studies included the establishment of on–boarding programs for newly hired physicians and organizational justice. Organizational justice refers to physicians’ perceptions of fairness in the workplace. On–boarding programs for physicians generally focus on four key aspects: credentialing and employment, orientation, marketing, and staff integration [22]. Around 29% of the studies (n = 2) listed management reforms as a policy intervention for addressing physician retention [22,23]. The advantages of these reforms included the potential retention of physicians in primary care and general practice, and improvement of physician morale and workplace environment. The disadvantage of this policy was the uncertainty in influencing intentions of new general practitioners [22,23].

## DISCUSSION

The studies included in this systematic review explored push and pull factors that affect physician movement from the public to the private sector. Six studies focused on low income and low middle income countries (lower income countries) and six studies focused on upper middle income and high–income countries (higher income countries) (**Table 1**). A country’s economic context influenced the factors affecting physicians’ choice of workplace. The influence of economic context was apparent in the variation in the number of studies that highlighted issues for each factor affecting physician’s choice of workplace (**Figure 1**).

In terms of factors, it is interesting to note that managerial interference, relationship with patients and income relative to workload were only reported in higher income countries. Similarly, working conditions, sustainability of income and education and training opportunities were only mentioned in lower income countries.

The most frequently reported theme across all the studies was financial incentives. In lower income countries the most recurring themes in descending order were: financial incentives, career development, infrastructure and staffing, workload, autonomy and professional work environment (**Figure 1**). In higher income countries these were: financial incentives, professional work environment, workload, autonomy, career development and infrastructure and staffing (**Figure 1**).

In terms of infrastructure and staffing, lack of resources such as drugs and equipment, poor working conditions, poor facility infrastructure, lack of clean water and electricity, lack of cleanliness in government facilities and staffing shortages were reported as push factors in Bangladesh, Pakistan, and Uganda [10,11,15]. In addition, staffing, workload and autonomy were important driving factors for physician’s choice of workplace in these lower income countries. In South Africa, a higher income country, the public sector was also found to have fewer resources and less equipment and drugs available, shortage in hospital staff, and poor working conditions compared to the private sector [3].

Career development was also one of the main key drivers for physicians to work in the private sector in higher income countries. Examples of the motivating factors that were listed in the studies include: recognition and prestige, the opportunity to work independently, the ability to apply their ideas, and/or the ability to provide individualized care [3,12,14,24]. However, in higher income countries like South Africa and New Zealand that have strong public health systems, there are greater opportunities for further education, and professional development in the public sector compared to the private sector [3,18].

In terms of workload, in higher income countries, tight inflexible schedules and heavy workload were reported as factors pushing physician out of the public sector in higher income countries [18,24]. Lower income countries also reported similar factors such as long duty hours and heavy workloads [10,11]. The professional work environment also plays a role in driving physicians from the public to the private sector. Generally, lack of respect and appreciation of physicians by human resources staff, high managerial interference and lack of trust toward the government were reported in higher income countries as factors driving physicians away from the public sector [3,20]. However, these factors were also reported in Uganda [10].

The review identified a range of policy interventions that focused on improving physician retention in the public sector. The country’s income category determined the type of policy interventions that was discussed. Regulatory controls and incentives were reported in both higher and lower income countries. Self–regulation and compulsory services were only reported in lower income countries. However, management reforms were only highlighted in higher income countries.

Regulatory interventions were among the most common policies used. Permitting dual practice was the most recurring policy intervention for both lower and higher income countries. Although there was no evidence to support compulsory services, self–regulation had some advantages such as establishing barriers for physicians to enter the private sector and addressing the uncontrolled proliferation of private physicians. There were no advantages reported for banning dual practice in both income settings.

The majority of the studies in lower and higher income countries reported using some type of incentive in addition to or instead of regulatory controls. Providing professional development opportunities was only reported in lower income countries and its advantages included improving physician competency and motivation [2]. Offering exclusive or rewarding contracts were highlighted as policy options in both lower and higher income countries. However, financial incentives were noted to be costly and posed a heavy burden on lower income countries.

Management reforms were specific to higher income countries; however, interventions such as supervisor trainings and on–boarding programs may increase physician retention in the public sector. As Heponiemi et al. reported that by enhancing organization justice, primary care facilities could improve general practitioners’ (GP) job satisfaction and potentially increase the attractiveness of GP work as a career option [22]. Organizational justice and effective on–boarding programs may help increase physician retention in the public sector, and reduce turnover by decreasing recruitment costs.

An interesting finding from this review is the high degree of similarity that exists between the push–pull factors that we identified and the brain drain issues that drive physicians to migrate both within and across countries. In a systematic review that aimed to examine motivating factors that would reduce medical migration both within and across countries, the following themes were identified: financial incentives, career development, continuing education, hospital infrastructure, resource availability, hospital management and personal recognition or appreciation [27]. These themes were all highlighted in our review; however, workload and autonomy seem to be specific to physicians’ choice of workplace between the public and private sector.

A major drawback in this review is the limited availability of published literature on physician retention in the public sector. Through the database search we only identified 19 studies that met our inclusion criteria, which might have impacted the interpretation of our findings as well as generalizability. Although we attempted to connect the variation in themes to different income levels, the sample size was too low to make any causal conclusions. Another factor affecting generalizability is the regional distribution of the studies, 53% of the studies focused on Sub–Saharan African and Asian countries and only 26% of studies were on North America, Oceania and Europe. There were no studies on Latin America and the Caribbean, Middle East and North Africa. In addition, only studies in English were included and other databases such as those for humanities and social sciences were not considered. However, the majority of physician studies would be expected to be cross–listed in medical databases examined here. Another limitation, is the difficulty of drawing conclusions from studies that were conducted in different countries, at different times, with different methodologies, and in different health systems and regulatory environments.

To the best of our knowledge this may be the first systematic review that specifically focuses on examining factors influencing physician retention and different policy interventions for improving retention in the public sector. Given the limited literature on physician retention in the public sector additional research is required, particularly to test the effectiveness of policy options for retention of physicians. It would also be useful for future cross–country research to use standardized data collection tools, allowing comparison of contextual factors as well as the examination of how context affects physician retention in the public sector. Given that financial incentives were frequently reported in both lower and higher income countries, cost controlling mechanisms for the private sector should be implemented such as benchmarking physician salaries with the public sector. The lack of private sector regulation in lower income countries as well as higher income countries needs to be addressed, this could be implemented as part of the efforts for expanding universal health coverage.
